# Supplementary material for: Portulaca oleracea L. Extract Regulates Hepatic Cholesterol Metabolism via the AMPK/MicroRNA-33/34a Pathway in Rats Fed a High-Cholesterol Diet
Source: Nutrients. 2022 Aug 14;14(16):3330. doi: 10.3390/nu14163330 (PMC9414803; doi:10.3390/nu14163330)
Supplement: Supplementary file 1 [file nutrients-14-03330-s001.zip › nutrients-1837054-supplementary.pdf]

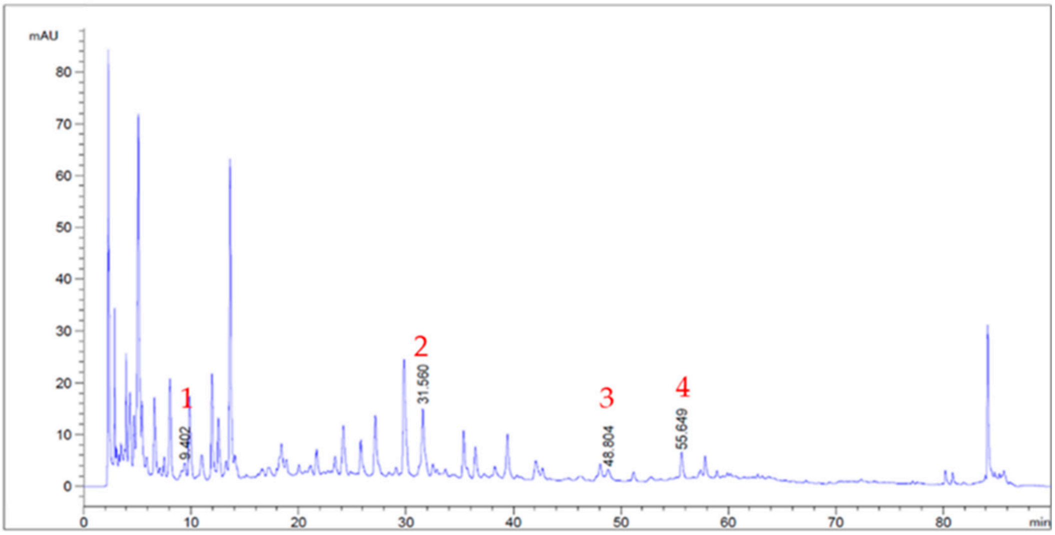

Figure S1. HPLC analysis of PE. Peak 1, gallic acid; Peak 2, chlorogenic acid; Peak 3, ferulic acid; Peak 4, rutin. PE, extruded *Portulaca oleracea* L. extract.

Table S1. Chromatographic parameters of phenolic compound standards analyzed by HPLC

Compound	Regression equation	r^2
Gallic acid	y=25.954x-220.916	0.999
Chlorogenic acid	y=16.830x-127.714	0.999
Ferulic acid	y=66.322x-143.433	0.999
Rutin	y=10.276x-7.209	0.999
